# Potential benefits of medium chain fatty acids in aging and neurodegenerative disease

**DOI:** 10.3389/fnagi.2023.1230467

**Published:** 2023-08-23

**Authors:** Ella Dunn, Biqin Zhang, Virender K. Sahota, Hrvoje Augustin

**Affiliations:** Department of Biological Sciences, Centre for Biomedical Sciences, Royal Holloway University of London, Egham, United Kingdom

**Keywords:** ageing, amyotrophic lateral sclerosis, Parkinson’ s disease, Alzheimer’s disease, medium chain fatty acid (MCFA), autophagy, mitochondria, ketogenic diet (KD)

## Abstract

Neurodegenerative diseases are a large class of neurological disorders characterized by progressive dysfunction and death of neurones. Examples include Alzheimer’s disease, Parkinson’s disease, frontotemporal dementia, and amyotrophic lateral sclerosis. Aging is the primary risk factor for neurodegeneration; individuals over 65 are more likely to suffer from a neurodegenerative disease, with prevalence increasing with age. As the population ages, the social and economic burden caused by these diseases will increase. Therefore, new therapies that address both aging and neurodegeneration are imperative. Ketogenic diets (KDs) are low carbohydrate, high-fat diets developed initially as an alternative treatment for epilepsy. The classic ketogenic diet provides energy via long-chain fatty acids (LCFAs); naturally occurring medium chain fatty acids (MCFAs), on the other hand, are the main components of the medium-chain triglyceride (MCT) ketogenic diet. MCT-based diets are more efficient at generating the ketone bodies that are used as a secondary energy source for neurones and astrocytes. However, ketone levels alone do not closely correlate with improved clinical symptoms. Recent findings suggest an alternative mode of action for the MCFAs, e.g., via improving mitochondrial biogenesis and glutamate receptor inhibition. MCFAs have been linked to the treatment of both aging and neurodegenerative disease via their effects on metabolism. Through action on multiple disease-related pathways, MCFAs are emerging as compounds with notable potential to promote healthy aging and ameliorate neurodegeneration. MCFAs have been shown to stimulate autophagy and restore mitochondrial function, which are found to be disrupted in aging and neurodegeneration. This review aims to provide insight into the metabolic benefits of MCFAs in neurodegenerative disease and healthy aging. We will discuss the use of MCFAs to combat dysregulation of autophagy and mitochondrial function in the context of “normal” aging, Parkinson’s disease, amyotrophic lateral sclerosis and Alzheimer’s disease.

## 1. Introduction

Neurodegeneration is defined as the progressive dysfunction, structural impairment, and eventual death of neuronal cells. Neurodegenerative diseases (NDs) are predominantly adult-onset pathologies that can have familial (genetic) or sporadic causes and form a large class of neurological disorders that are the main cause of disability and the second leading cause of death worldwide (Erkkinen et al., 2018; Bannick et al., 2019). Globally, the most common NDs are Alzheimer’s disease (AD), Parkinson’s disease (PD), and amyotrophic lateral sclerosis (ALS).

The ketogenic diet is a low-carbohydrate, high-fat diet. The classic KD utilizes long chain triglycerides (LCTs)–consisting of long chain fatty acids (LCFAs)–to provide between 60 and 80% of dietary energy. The alternative type of ketogenic diet relies on medium chain triglycerides (MCTs) that are hydrolyzed to medium chain fatty acids (MCFAs) as a primary energy source (Neal, 2017). LCFAs and MCFAs differ by the number of carbon atoms: LCFAs have between 16 and 20 carbon atoms, whereas MCFAs have 6–12 (Neal, 2017; Table 1). After hydrolysis of triglycerides to fatty acids in the gut, ketone bodies (known as ketones) are generated in the liver via β-oxidation. Compared to LCFAs, MCFAs are more rapidly metabolized and generate ketones more efficiently, allowing for a more palatable diet due to the higher carbohydrate content (Huttenlocher et al., 1971).

**TABLE 1:** Medium chain fatty acids.

IUPAC name	Common name	Carbon atoms	Chemical formula	Molecular structure
Hexanoic acid	Caproic acid	6	CH_3_(CH_2_)_4_COOH	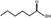
Heptanoic acid	Enanthic acid	7	CH_3_(CH_2_)_5_COOH	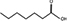
Octanoic acid	Caprylic acid	8	CH_3_(CH_2_)_6_COOH	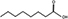
Nonanoic acid	Pelargonic acid	9	CH_3_(CH_2_)_7_COOH	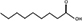
Decanoic acid	Capric acid	10	CH_3_(CH_2_)_8_COOH	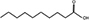
Undecanoic acid	Undecylic acid	11	CH_3_(CH_2_)_9_COOH	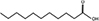
Dodecanoic acid	Lauric acid	12	CH_3_(CH_2_)_10_COOH	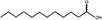

For almost a century, the ketogenic diet has been used in the treatment of drug-resistant epilepsy (Martin et al., 2016). Recent *in vitro* and *in vivo* studies suggest a wider, neuroprotective role of the KD in the context of cancer, neurodegenerative diseases, and normal, healthy aging (Moreno and Mobbs, 2017). The mechanisms of action of the KD are not fully understood but are likely related to improved mitochondrial function and energy metabolism, and enhanced autophagy.

In this review, we discuss how impaired autophagy and mitochondrial dynamics affect aging and pathogenesis of NDs, focusing on AD, PD, and ALS, and provide an overview of experimental evidence suggesting novel therapeutic avenues aimed at exploiting the beneficial effects of MCFAs on these processes (Table 2).

**TABLE 2:** The potential effects of medium chain fatty acids (MCFAs) on autophagy and mitochondrial function in aging and neurodegenerative diseases.

MCFA	Effects on autophagy and mitochondrial dysfunction in:				References
	Aging	Alzheimer’s disease (AD)	Amyotrophic lateral sclerosis (ALS)	Parkinson’s disease (PD)	
Hexanoic acid	No literature available				
Heptanoic acid	No literature available				
Octanoic acid	Stimulates JNK-dependent autophagy, known to extend the lifespan in *Drosophila*	–	–	–	Wang et al., 2005; He et al., 2022
Nonanoic acid	–	–	–	–	
Decanoic acid	Increases autophagy by *Atg1* and *Atg8a* upregulation. Reduced autophagy contributes to functional decline in aging. Reduces oxidative stress, increase mitochondria biogenesis, and upregulate mitochondrial respiratory chain enzymes via activation of PPARγ, SIRT1 and SIRT3. Elevated oxidative stress and reduced mitochondrial function contribute to functional decline in aging.	Reduces excitotoxicity by AMPAR inhibition. Over-excitation of the AMPAR contributes to AD.	Decreases autophagy by inhibiting mTORC1 in patient-derived astrocytes. mTORC1 inhibition was previously shown to improve locomotion in zebrafish ALS model. Reduces excitotoxicity by AMPAR inhibition. Over-excitation of the AMPAR contributes to ALS.	Reduces excitotoxicity by AMPAR inhibition. Over-excitation of the AMPAR contributes to PD. Reduces dopaminergic neuron loss and oxidative stress via activation of SIRT3. Reduced SIRT3 was reported with dopaminergic neuron loss and increased oxidative stress in the PD mouse model.	Johnson et al., 2009; Van Damme, 2009; Malapaka et al., 2012; Hughes et al., 2014; Joshi et al., 2015; Lattante et al., 2015; Liu et al., 2015; Chang et al., 2016; Mesquita et al., 2017; Whitehead et al., 2017; Jurado, 2018; Dabke and Das, 2020; Warren et al., 2020, 2021; Akamatsu et al., 2022
Undecanoic acid	No literature available				
Dodecanoic acid	–	–	–	Reduces augmented autophagy by decreasing Atg5 and Beclin-1. *LRRK2* mutations increase autophagy, resulting in neurite shortening which precedes neuronal death.	Plowey et al., 2008; Sekar et al., 2018
Medium-chain triglyceride (MCT) ketogenic diet, consisting of various MCFAs	Nutritional ketosis upregulates autophagy by inhibiting mTORC1. Reduced autophagy contributes to functional decline in aging. Upregulates mitochondrial respiratory chain enzymes. Promotes autophagy and mitochondrial biogenesis via AMPK and PGC-1α activation.	Ketone-dependent autophagy regulation of HMGS2, an enzyme controlling ketone synthesis from MCFAs, reduces amyloid-β plaques	Promotes mitochondrial ATP synthesis and prevents complex I inhibition.	Ketones generated from MCFA metabolism directly promote chaperone-mediated autophagy (CMA). Activation of CMA induces LRRK2 and α-synuclein degradation. Improves mitochondrial respiration by reducing glutamate- induced ROS. ROS production induced by excitotoxicity of glutaminergic neurones was observed in PD	Cuervo and Dice, 2000; Finn and Dice, 2005; Zhao et al., 2006; Jäger et al., 2007; Maalouf et al., 2007; Egan et al., 2011; McDaniel et al., 2011; Orenstein et al., 2013; McCarty et al., 2015; Hu et al., 2017; Dabke and Das, 2020

## 2. MCT-based diets, neurodegenerative diseases, and aging

### 2.1. Neurodegenerative diseases and “healthy” aging

Multiple disease-causing factors have been identified for AD, PD, and ALS, ranging from environmental to genetic. A pathological feature shared by all three NDs is the accumulation of protein inclusions that are believed to play a critical role in the onset of neurodegeneration (Longhena et al., 2017). AD is the most common ND, causing progressively severe and irreversible cognitive and physical decline. The major hallmarks of AD pathology are accumulation of β-amyloid plaques in the brain, and the hyperphosphorylation and aggregation of tau protein to form neurofibrillary tangles, both of which trigger neuronal cell death (Luque and Jaffe, 2009). The loss of dopaminergic neurones and the accumulation of Lewy bodies (LB), cytoplasmic inclusions consisting of α -synuclein in the brain, leads to PD, the fastest growing neurological disorder in the world (Volpicelli-Daley et al., 2014; Poewe et al., 2017; Raza et al., 2019). ALS, the most common form of motor neurone disease (MND), is characterized by a progressive, paralysis-causing loss of motor neurones in the brain and spinal cord (Hulisz, 2018) and the presence of inclusions containing TDP-43, a protein that becomes pathological when hyper-phosphorylated or hyper-ubiquinated (Lillo and Hodges, 2009). At the molecular level, human NDs are characterized by proteotoxic stress, oxidative stress, neuroinflammation, mitochondrial dysfunction, excitotoxicity and autophagy dysregulation (Dong et al., 2009; Dugger and Dickson, 2017). The convergence of biological processes implicated in different NDs indicates common mechanisms driving neurodegeneration. Currently, there are no treatments able to sufficiently alter any ND, signifying a need for further research and funding.

The greatest risk factor for all major NDs is aging. Biological aging is described as the gradual functional decline of cells, tissues, and organs, leading to physical, physiological, metabolic and psychological changes over time and increased vulnerability to death. The molecular and cellular processes underlying aging including genomic instability, telomere loss, epigenetic changes, compromised protein homeostasis, deregulated nutrient sensing, mitochondrial dysfunction, cellular senescence, stem cell exhaustion and altered intercellular communication (López-Otín et al., 2013). A large body of experimental evidence suggests that amelioration of these hallmarks of aging may extend lifespan and improve the health status in various animal models of “healthy” aging and human diseases, prompting the search for genetic, pharmacological and dietary modifiers of these processes. Due to their ability to modulate many of the cellular mechanisms underlying aging and pathobiology of NDs, MCFAs emerged as a promising class of molecules for intervention into these processes.

### 2.2. MCT-based diet

The MCT diet relies on three main ketones to elicit metabolic effects: acetone, acetoacetate and β-hydroxybutyrate (BHB) (Schonfeld and Wojtczak, 2016). Ketone-based diets are known to modulate levels of GABA and glutamate, inhibit voltage dependent calcium channel regulation and alter mitochondrial function and ATP availability (Bough and Rho, 2007; Lutas and Yellen, 2013; Kadowaki et al., 2017). Under normal dietary conditions, glucose is utilized as a main energy source. In conditions where glucose is unavailable, such as in fasting conditions, ketones become the main energy source. Whereas ketone bodies can be neuroprotective (Kim et al., 2007; Yang et al., 2019), there is a lack of clear correlation between plasma ketone levels in mouse seizure models (Tan et al., 2017), and *ex vivo* studies indicate that fatty acids, rather than ketones, provide anti-seizure activity in rat models of epilepsy (Chang et al., 2016). These experimental data suggest the existence of additional modes of action through which ketogenic diets can exert their beneficial effects on the nervous system.

Octanoic acid and decanoic acid are medium chain fatty acids (MCFAs; Table 1) and components of the medium chain triglyceride (MCT) ketogenic diet. These MCFAs can provide an alternate energy source to glucose for neurones and astrocytes (Cunnane et al., 2020). NDs are associated with metabolic dysfunction, specifically with altered glucose and lipid metabolism in the brain, indicating MCFAs as a potential treatment option (Wlaz et al., 2012; Cunnane et al., 2020; Estes et al., 2021). Importantly, recent findings demonstrate a pharmacological mode of action for MCFAs through its inhibitory action on α-amino-3-hydroxy-5-methyl-4-isoxazolepropionic acid (AMPA)-type glutamate receptors (Chang et al., 2016) and activation of the peroxisomal proliferator-activated receptor gamma (PPARγ) (Hughes et al., 2014).

Medium chain fatty acids and the MCT diet improve cognitive performance of patients suffering from AD and mild cognitive impairment (Krikorian et al., 2012; Taylor et al., 2018). In addition, MCFAs have been shown to improve mitochondrial function and promote autophagy, both of which are known to be dysregulated in aging and NDs (Rangaraju et al., 2019; Stavoe and Holzbaur, 2019).

Importantly, MCFAs can cross the blood brain barrier (BBB) (Wlaz et al., 2012, 2015), raising the possibility that MCFAs may also be used to treat conditions affecting the central nervous system, including AD, ALS, PD, and even some types of brain tumors (Castellano et al., 2015; Croteau et al., 2017; Tefera et al., 2017; Manzo et al., 2018; Zeng et al., 2019). The ability of MCFAs to traverse the BBB provides an advantage from a pharmacological perspective, especially as drug delivery to the nervous system can be problematic. MCFAs can be administered via multiple routes. Patients can adhere to the MCT diet by restricting carbohydrates and eating foods that supply them with the specific MCFAs (Grammatikopoulou et al., 2020). Ready-to-consume drink supplements are available, as well as pre-prepared ketogenic formulas and meals (Schoeler et al., 2021); patients may also choose to supplement their diet with capsules and oils containing the MCFAs they require (Grammatikopoulou et al., 2020).

### 2.3. Current uses of MCT-based diets, preclinical studies, and caveats

The MCT diet was originally developed as a treatment for epilepsy as starvation, a condition in which ketones are utilized as the main energy source, had long been observed to reduce the frequency of seizures (Martin et al., 2016). For patients that have developed resistance to the standard, pharmacological epilepsy treatments, the MCT diet is especially important as it provides an alternative treatment option (Loscher et al., 2020). Furthermore, an MCT diet containing only decanoic and octanoic acids has been shown to improve attention and structural connectivity in the brains of patients with mild cognitive impairment (Roy et al., 2022).

In pre-clinical trials, the classic ketogenic diet has been proven beneficial in the treatment of multiple diseases and disorders. For example, ketone-producing diet can prolong life and reduce seizures in mouse models of Dravet syndrome, an early onset childhood developmental disorder with severe epileptic encephalopathy (Jancovski et al., 2021). A reduction in anxiety is seen in rats on the MCT diet, along with an increase in social competitiveness (Hollis et al., 2018). The ketogenic diet is under assessment for treating multiple sclerosis, as a 3-day fasting cycle in which ketones are used as a primary source of energy appears to alleviate symptoms associated with MS-related autoimmunity (Choi et al., 2016). In mice, a ketogenic diet suppresses insulin resistance and inflammation in a high fat diet-induced model of obesity (Geng et al., 2016). The ketogenic diet is also beneficial in Duchenne muscular dystrophy, where muscle function was restored in a rat model (Fujikura et al., 2021). An oil-based MCT diet has also been used to treat hypertrophic cardiomyopathy in a patient with a deficiency in the gene coding for acyl-coA dehydrogenase (Pervaiz et al., 2011).

Although both OA and decanoic acid (DA) constitute the main fatty acids in the MCT-diet, they appear to have distinct roles. The effect of DA on mitochondrial function appears to be stronger when compared to OA (discussed later). In the brain, β-oxidation of OA is favored, whereas DA preferentially stimulates lactate production through glycolysis which is then utilized as an energy source by brain cells. β-oxidation of both OA and DA occurs through the action of carnitine palmitoyltransferase, (CPT) but it is thought that OA also undergoes oxidation that is independent of CPT (Khabbush et al., 2017). CPT levels in neurones is low, which may explain why DA is spared from oxidation and appears to be present at higher levels than OA.

Despite their advantages and clinical benefits, ketogenic diets should be administered with caution. As they mimic starvation conditions, patients must be very carefully monitored to avoid possible side effects such as acidosis and hypoglycemia (Sankar and Sotero de Menezes, 1999). Ketogenic dietary regimens are known to result in weight loss and can be very effective to help patients with obesity reach a healthy weight. However, if prescribed to an individual with a healthy weight, extra weight loss could be detrimental to the patients’ health (Bueno et al., 2013). The ketogenic diet is not recommended for patients with liver failure, porphyria, pyruvate kinase deficiency, carnitine translocase deficiency, primary carnitine deficiency, pancreatitis, and carnitine palmitoyl transferase deficiency (Maswood et al., 2020). The ketogenic diet also comes with a range of short-term side effects, commonly referred to as the “keto flu,” including fatigue, dizziness, nausea, vomiting and headaches (Maswood et al., 2020). Short-term symptoms usually subside when the body has adjusted to the diet, however, long-term side effects such as the build-up of kidney stones are not uncommon; in addition, most patients will require supplementation to prevent vitamin deficiencies (Wilong, 2007; Maswood et al., 2020). Recent clinical findings demonstrate a correlation between a long-term ketogenic diet and reduced bone density (Simm et al., 2017) and experiments in mice suggest a significant negative effect on bone health by octanoic acid, a major component of the MCT diet (Jain et al., 2021). Overall, following a ketogenic or MCT-based diet has the potential to compromise the nutritional status of some patients necessitating regular monitoring and nutrient supplementation.

Another important consideration is that some ketogenic diets are very hard to follow. Due to their restrictiveness, patients are limited on what they can eat, and compliance issues are very prominent. The retention rate can be partially improved by enhancing the taste of pre-prepared foods and increasing patient support (Tong et al., 2022).

## 3. Mitochondrial dysfunction in aging and neurodegenerative diseases

**FIGURE 1:**
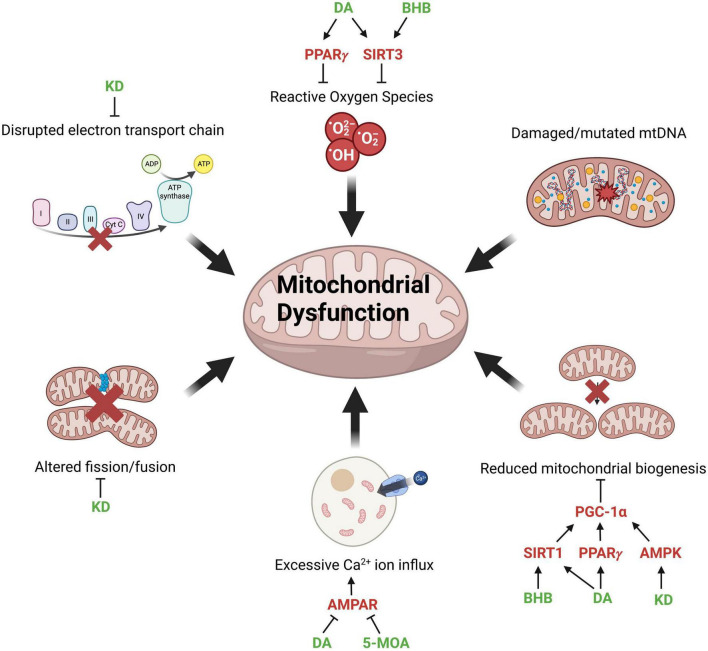
Factors contributing to mitochondrial dysfunction and potential points of intervention. Mitochondrial dysfunction associated with aging and neurodegenerative diseases and molecular modulators of mitochondrial function (red) through which MCFAs (medium-chain fatty acids), MCT (medium-chain triglyceride) diet or KD (ketogenic diet) (green) can potentially reverse the pathological phenotype. The production of ROSs (reactive oxygen species) is mitigated by DA (decanoic acid) and BHB (β-hydroxybutyrate) activating PPARγ (peroxisome proliferator-activated receptors gamma) and SIRT3 (sirtuin 3). Reduced mitochondrial biogenesis is restored by BHB, DA, and KD through the activation of SIRT1 (sirtuin 1), PPARγ, and AMPK (AMP-activated protein kinase), which subsequently activate PGC-1α (PPARγ coactivator 1-alpha) to promote mitochondrial biogenesis. Excessive calcium influx into cells is reduced by DA and 5-MOA (5-methyl octanoic acid), which inhibit AMPAR (AMPA-type glutamate receptors). KD reverses the disrupted mitochondrial dynamics and ETC (electron transport chain) function via inhibition of Drp1 (dynamin-related protein 1) and enhances the expression of ETC complexes, respectively. Created with BioRender.com.

Mitochondria are responsible for generating most of the adenosine triphosphate (ATP) supply in the cell through oxidative respiration (Mookerjee et al., 2010). ATP production in mitochondria is achieved by transferring electrons from NADH and FADH_2_ generated by glycolysis and the Krebs cycle through Complexes I to IV of the Electron Transport Chain (ETC) (Martínez-Reyes and Chandel, 2020). Complex I, III, and IV use high energy electrons to pump protons to the intermembrane space creating a proton gradient across the inner mitochondria membrane. The transfer of protons back to the mitochondria matrix drives the production of ATP via Complex V (Zhao et al., 2019). Interestingly, muscle and brain tissues of aged mice fed a ketogenic diet exhibited increased levels of the Krebs cycle protein citrate synthase and Complex I and IV proteins (Zhou et al., 2021; Figure 1).

Mitochondrial dysfunction is a hallmark of both NDs and normal, “healthy” aging (Figure 1). As an organism ages, mitochondrial ATP production declines due to increased electron leakage and consequent uncoupling of the ETC (Mookerjee et al., 2010). The brain requires a large amount of energy and neuronal cells have a limited glycolytic capability, making them extremely reliant on the energy produced by mitochondria and vulnerable to mitochondrial dysfunction (Moreira et al., 2009). Treatments that can improve mitochondrial output may therefore be beneficial in rescuing symptoms of aging and NDs. Mitochondria are dynamic and constantly undergo fission and fusion (Zhu et al., 2013). However, in AD, ALS, and PD, mitochondrial dynamics is disrupted, resulting in mitochondrial fragmentation and reduced energy output (Wang et al., 2009; Lim et al., 2012; Jiang et al., 2015). Mitochondrial fission is regulated by the dynamin-related protein 1 (Drp1). Interestingly, KD has been shown to promote mitochondrial integrity by suppressing the mitochondrial translocation of Drp1 and reducing ER stress (Guo et al., 2018; Figure 1). On the other hand, although damaged or mutated mtDNA contributes to mitochondrial dysfunction, there is minimal evidence suggesting that MCFAs are directly beneficial in restoring the structural integrity of mtDNA.

### 3.1. MCFAs may promote healthy aging by upregulating mitochondrial biogenesis and reducing oxidative stress

The 10-carbon DA can activate pathways or receptors related to mitochondrial function, such as peroxisome proliferator-activated receptors (PPARs) (Michalik et al., 2006; Malapaka et al., 2012). PPARs are involved in various physiological processes, including energy metabolism and inflammation (Varga et al., 2011). Mice fed with PPARγ agonists exhibited extended lifespan, improved cognitive ability, reduced inflammation, and enhanced mitochondrial function (Xu et al., 2020). Decanoic acid is identified as a modulator that directly binds and activates PPARγ (Malapaka et al., 2012). Moreover, DA stimulates complex I activity and increases mitochondrial number in cultured neurones through the regulation of PPARγ (Hughes et al., 2014; Figure 1).

Sirtuins are a family of proteins involved in stress response, epigenetic modification, cellular metabolism and longevity (Houtkooper et al., 2012). Three of the seven known sirtuins, SIRT3, SIRT 4, and SIRT5 are predominantly expressed in the mitochondria and believed to function as a link between metabolism and aging (Ji et al., 2022). *SIRT1* and *SIRT3* display a reduced expression with age (Donmez and Guarente, 2010; Jing et al., 2013), resulting in increased oxidative stress and reduced mitochondrial biogenesis due to reduced activation of PGC-1α (PPARγ coactivator 1-alpha), a key regulator of mitochondrial biogenesis (Onyango et al., 2002; Nemoto et al., 2005). Interestingly, cultured hippocampal neurones treated with BHB or DA show activation of SIRT1 and SIRT3, as well as upregulation of mitochondrial respiratory chain enzymes (Dabke and Das, 2020; Figure 1 and Table 2).

The AMP-activated protein kinase (AMPK), a critical sensor of intracellular ATP levels and regulator of energy homeostasis, is another protein known to decline with age (Reznick et al., 2007; Hardie et al., 2012; Herzig and Shaw, 2018). Age-related decline of AMPK results in reduced activation of PGC-1α, with a negative effect on mitochondrial biogenesis (Jäger et al., 2007; Figure 1) and autophagy (Egan et al., 2011). Little is known regarding the effect of MCFAs on AMPK activity, however, rats with *ad libitum* access to KD showed an elevated level of AMPK signaling in the liver (McDaniel et al., 2011), warranting further evaluation of the role of MCT-based diets in modulating this pathway.

### 3.2. MCFAs may improve symptoms of Alzheimer’s disease by promoting mitochondrial biogenesis

Multiple studies have linked mitochondrial dysfunction to Alzheimer’s disease (AD). PGC-1α, a crucial regulator of mitochondrial biogenesis, is suppressed in both AD models and patients (Qin et al., 2009; Sheng et al., 2012); *SIRT1* expression and activity are also found to be reduced in the brains of AD patients (Julien et al., 2009; Lalla and Donmez, 2013). Reduced PGC-1 activation and downregulation of *SIRT1* also promote amyloid-beta (Aβ) production, impaired Aβ clearance and tau hyperphosphorylation, resulting in the formation of Aβ plaques and neurofibrillary tangles (Qin et al., 2006; Min et al., 2010). The accumulation of Aβ has been shown to affect mitochondrial bioenergetics, dynamics, distribution and clearance in human cell cultures, yeast, fruit fly and mice (Iijima-Ando et al., 2009; Sinclair et al., 2021; Lee et al., 2022; Epremyan et al., 2023; Zyśk et al., 2023). Altered glucose metabolism is one of the hallmarks of AD (Hoyer, 1991; Schubert, 2005; Calsolaro and Edison, 2016). Due to its positive effect on glucose levels, KD has been proposed as a potential treatment for AD (Broom et al., 2019). Furthermore, KD-induced elevated ketones have been reported to have a neuroprotective effect by reducing oxidative stress in AD and PD models (Kashiwaya et al., 2000; Kim et al., 2007; Yang et al., 2019). Indeed, MCT diet improved cognitive function in AD patients with mild to moderate symptoms of AD (Ota et al., 2019). Another study also showed that patients who followed the MCT diet had an improved brain energy metabolism (Croteau et al., 2018). Although the underlying mechanisms remain unclear, it was proposed that the beneficial effect of the MCT diet on AD stems from the ability of the ketones to provide an alternative fuel source to the brain cells that are damaged or impaired due to metabolic dysfunction (Takeishi et al., 2021). Ketones in the brain are also shown to reduce inflammation and oxidative stress, two known contributors to the development of AD (Pinto et al., 2018). In addition, the brains of AD patients exhibit altered levels of mitochondrial respiratory complexes, along with impaired axonal transport and synaptic morphology, and increased ROS production and oxidative stress (Manczak et al., 2004; Rice et al., 2014; Pickett et al., 2018; Butterfield and Halliwell, 2019).

Synaptic dysfunction is one of the early signs of AD and other neurodegenerative diseases (Taoufik et al., 2018). AMPA-type glutamate receptors (AMPARs) are Ca^2+^ permeable channels that mediate excitatory synaptic transmission in the brain and contribute to glutamate receptor-mediated neurodegeneration (Greig et al., 2000; Palmer et al., 2005; Joshi et al., 2011). Over-excitation of the AMPAR leads to excitotoxicity, which is implicated in AD, ALS and PD (Johnson et al., 2009; Van Damme, 2009; Joshi et al., 2015; Whitehead et al., 2017; Jurado, 2018; Akamatsu et al., 2022), leading to the idea that inhibition of AMPARs could potentially improve the symptoms and progression of AD and other NDs. A recent study identified DA as a selective inhibitor of excitatory synapses in hippocampal slices and a non-competitive antagonist of AMPA receptors, explaining its anti-convulsant effect in the *in vitro* seizure model (Chang et al., 2016). OA, another key MCT diet component (Masino, 2022), does not inhibit AMPARs (Chang et al., 2016). However, its modified version, 5-methyl octanoic acid (5-MOA), inhibits AMPARs both *in vitro* and *in vivo*, suggesting potential novel therapeutic interventions for AD (Chang et al., 2015; Figure 1).

### 3.3. MCFAs could ameliorate symptoms of amyotrophic lateral sclerosis via improved mitochondrial biogenesis

The exact molecular mechanisms behind motor neurone degeneration in ALS are currently unclear, however, there is increasing evidence implicating mitochondrial dysfunction in the pathogenesis of ALS (Zhao et al., 2022). Aggregation of swollen mitochondria in the neurones of ALS patients was one of the first pathological changes seen in the disease, providing clear evidence of dysfunction (Atsumi, 1981; Taylor et al., 2016). Morphologically abnormal mitochondria have been observed in multiple cellular and animal models of ALS; mitochondria appear fragmented, vacuolated and more spherical, and are seen in atypical clusters along the axon (Higgins et al., 2003; Magrané et al., 2014).

Defective mitochondrial respiration, ATP production and oxidative phosphorylation have also been widely reported in ALS patients. Indeed, post-mortem analysis of sporadic ALS patients demonstrated reduced complex I-IV activity in the spinal cord, and impairment of I and IV activity in skeletal muscle, leading to a reduction in ATP production (Wiedemann et al., 1998, 2002). Elevated ROS production and ROS-associated damage are common pathological features of ALS. Both post-mortem and biofluid analysis of ALS patients has demonstrated markers of ROS damage (Shaw et al., 1995; Smith et al., 1998). It is thought that oxidative damage via an increase in ROS production results in aggregation of TDP-43, one of the main pathological hallmarks in ALS (Cohen et al., 2012).

Amyotrophic lateral sclerosis mouse models subjected to the ketogenic diet have larger numbers of motor neurones in the spinal cord compared to mice on a normal diet (Zhao et al., 2006). In addition, motor performance of the “KD mice” was significantly enhanced due to preservation of motor neurones in the spinal cord. The same study detected a higher concentration of BHB (a ketone generated via MCFA metabolism) in the blood of KD-fed mice (Zhao et al., 2006). BHB was able to promote mitochondrial ATP synthesis and prevent the inhibition of complex I *in vitro*, likely contributing to the increased motor performance observed in the KD-treated ALS model mice (Zhao et al., 2006; Table 2). Dietary supplementation of OA, a key component of the MCT diet, had no effect on the survival rate of ALS mice (Zhao et al., 2012), but significantly improved motor performance, by protecting against spinal cord motor neurone loss (Zhao et al., 2012). This study concluded that OA treatment significantly promoted oxygen consumption rate, restoring energy metabolism (Zhao et al., 2012).

### 3.4. MCFAs may alleviate symptoms of Parkinson’s disease by elevating ketone levels

Mitochondrial dysfunction has long been associated with PD (Mullin and Schapira, 2013; Park et al., 2018). Mutations in the Leucine Rich Repeat Kinase 2 (LRRK2) gene are a common genetic cause of late-onset familial and sporadic PD (Kumari and Tan, 2009). The G2019S mutation increases LRRK2 kinase activity (Chen and Wu, 2018). Overexpression of either wild-type *LRRK2* or *LRRK2^*G*2019*S*^* leads to increased ROS production and reduced mitochondrial function in mice, fruit flies, iPSC cell cultures and PD patients (Mortiboys et al., 2010; Cooper et al., 2012; Ng et al., 2012; Yue et al., 2015). SIRT3, capable of reducing oxidative stress (Nemoto et al., 2005), is also decreased in the mouse model of AD (Yang et al., 2015). The formation of Lewy bodies, composed of aggregated α-Synuclein (α-Syn) protein, is another PD hallmark. Mutations in the *SNCA* gene, encoding α-Syn, induce ROS production and mitochondrial fragmentation (Ryan et al., 2015). α-Syn is located on mitochondria-associated membranes (MAM), which regulate Ca^2+^ signaling and apoptosis (Guardia-Laguarta et al., 2014). Excessive α-Syn disrupts mitochondria-ER interactions, impairing ATP output and Ca^2+^ exchange (Paillusson et al., 2017). α-Syn also suppresses mitochondrial biogenesis through PGC-1α inhibition (Ryan et al., 2013). Interestingly, PD patients exhibit under-expression of *PGC-1*α, resulting in the loss of dopaminergic neurones (Zheng et al., 2010).

Decanoic acid has been shown to reduce the degeneration of dopaminergic neurons in the *C. elegans* PD model by modulating the insulin signaling pathway—a critical regulator of longevity in both invertebrates and vertebrates—resulting in the enhanced transcription of genes encoding antioxidant and heat-shock proteins (Sanguanphun et al., 2022). Interestingly, the polyphenolic compound resveratrol has been shown to have an antioxidant effect by acting as a free radical scavenger, providing a neuroprotective effect via binding and activating SIRT1 (Iuga et al., 2012; Cao et al., 2015; Salehi et al., 2018). Resveratrol can also protect neuronal cells against oxidative stress and toxicity specifically induced by α-synuclein via SIRT1 activation (Albani et al., 2009). In line with these studies, SIRT3 is required to reduce dopaminergic neuron loss in a mouse model of PD, enhances mitochondrial antioxidant capacity and reduces oxidative stress (Liu et al., 2015). Although few studies directly investigated the effect of MCFAs on PD, multiple reports provide indirect evidence supporting MCFAs’ beneficial effect on PD via elevating the level of ketone bodies. Similar to AD, the KD has been shown to alleviate symptoms of PD (Kashiwaya et al., 2000). In one study, rat neuronal cells were treated with ketone bodies, specifically beta-hydroxybutyrate (BHB) and acetoacetate, resulting in decreased production of glutamate-induced ROS. This reduction was due to an increase in the NAD^+^/NADH ratio, which in turn led to improved mitochondrial respiration (Maalouf et al., 2007; Table 2). Previous study also showed enhanced mitochondrial respiration and a reduction of PD symptoms in PD model mice treated with D-BHB through subcutaneous injections (Tieu et al., 2003; Table 2). These findings suggest that the use of MCFAs may have potential therapeutic benefits in the treatment of PD.

Overall, MCFAs have shown potential in treating NDs and functional decline caused by aging via restoring mitochondrial function through the activation of the PPARγ and the inhibition of the AMPAR.

## 4. Autophagy in aging and neurodegenerative disease

**FIGURE 2:**
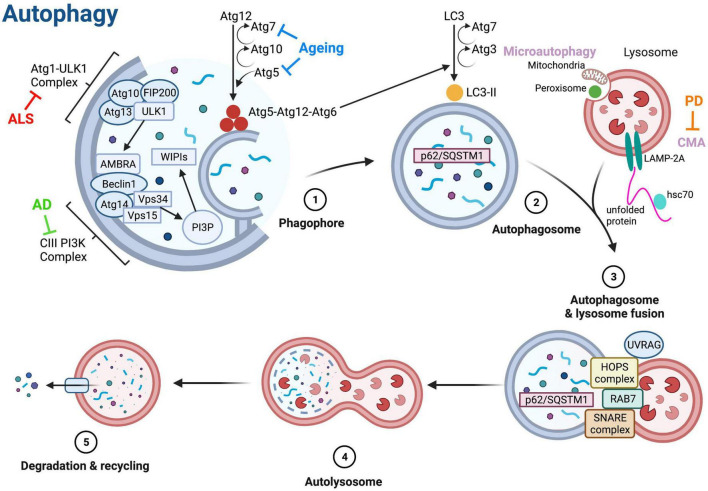
Molecular regulation of autophagy. The three main types of autophagy are macroautophagy (referred to as autophagy), microautophagy and chaperone-mediated autophagy (CMA). Autophagy is initiated via the autophagy-related (Atg) 1-Unc51-like kinase (ULK) complex that includes ULK1, Atg13, FIP200 (focal adhesion kinase family interacting protein of 200 kD) and Atg101. Phosphorylation of ULK1 stimulates the translocation of the class III phosphoinositide 3-kinase (PI3K-III) complex containing Beclin-1 from the cytoskeleton to a pre-autophagosomal structure, initiating the formation of the autophagosome (Suzuki et al., 2007; Mizushima, 2009; Di Bartolomeo et al., 2010). The activity of the PI3K-III complex generates phosphatidylinositol-3-phosphate (PI3P), which binds to the WD repeat domain phosphoinositide-interacting 1 (WIPI1) and WIPI2 complexes, catalyzing two ubiquitin-like reactions that allow elongation of the autophagosome membrane (Ohsumi and Mizushima, 2004). Atg5 and Atg12 are conjugated together in the first reaction, in the presence of Atg7 and Atg10 (Ohsumi et al., 2000). During the second reaction, a complex containing Atg5, Atg12, and Atg16 attaches to the membrane and induces the conjugation of phosphatidylethanolamine to LC3 (microtubule-associated protein 1A/1B-light chain 3), forming LC3-II and facilitating membrane closure (Fujita et al., 2008). LC3-II binds to autophagy receptors, such as Sequestosome 1 (p62/SQSTM1), that are bound to components targeted for degradation (Pankiv et al., 2007). Once autophagosomes are fully formed, the UV radiation resistance gene (UVRAG) protein is phosphorylated and combines with the homotypic fusion and protein sorting (HOPS) complex to assist with membrane trafficking and fusion, thus allowing fusion of the autophagosome with a lysosome (Liu and Sabatini, 2020). Ras-associated binding (Rab) proteins and soluble N-ethylmaleimide-sensitive-factor attachment protein receptor (SNARE) proteins also assist in fusion of autophagosomes and lysosomes (Aman et al., 2021). After fusion, the lysosomal content is degraded by lysosomal hydrolases, and degradation products are released into the cell for re-use (Aman et al., 2021). Microautophagy involves degradation of peroxisomes and mitochondria via invagination of the lysosome membrane (Kunz et al., 2004). In CMA, protein substrates are unfolded by heat shock cognate 71 kDa protein (hsc70) chaperones and translocated across the lysosome membrane via interaction with lysosome-associated membrane protein 2A (LAMP-2A) (Majeski and Fred Dice, 2004). Blunt arrows indicate main proteins/processes downregulated in ageing (blue), amyotrophic lateral sclerosis (ALS, red) and Alzheimer’s disease (AD, green), and Parkinson’s disease (PD, orange). Created with BioRender.com.

Dysfunctional protein homeostasis is a key feature of both aging and NDs, resulting in the accumulation of misfolded, mislocalized and aggregated proteins within neurones (Aman et al., 2021). There are two major pathways responsible for degradation of cellular proteins and maintenance of homeostasis: the ubiquitin proteasome system (UPS) and the autophagy-lysosome pathway (Lilienbaum, 2013). Normally, around 90% of cellular proteins throughout the body are degraded by the UPS, however, when the UPS is defective, autophagy can be upregulated to help clear larger protein aggregates (Rock et al., 1994; Kageyama et al., 2014). Autophagy is thought to be the primary method of protein clearance in ND patient neurones, as the UPS is unable to degrade the large pathological aggregates of proteins (Chandran and Rochet, 2022). Autophagy was first described in the 1960s as a lysosome-dependent pathway for degradation of damaged or unnecessary cellular components (De Duve and Wattiaux, 1966). There are three main types of autophagy: macroautophagy, chaperone mediated autophagy (CMA) and microautophagy (Figure 2). In microautophagy, cellular components requiring degradation are taken up directly by the lysosome through invagination of the membrane, whereas in CMA, proteins targeted for degradation are translocated across the lysosomal membrane with the help of a chaperone protein (Figure 2; Glick et al., 2010). Macroautophagy (hereafter referred to as autophagy) involves the delivery of cytoplasmic components to lysosomes for degradation via an autophagosome—an intermediary vesicle that binds to and fuses with the lysosome to form an autolysosome (Figure 2; Glick et al., 2010).

Autophagy is an intricate, multi-stage process; the main stages and critical proteins mediating autophagy are illustrated in Figure 2. Autophagic clearance is initiated via the autophagy-related 1 (Atg1)-Unc51-like kinase (ULK) complex; ULK1 phosphorylation via the activation of AMP-activated protein kinase (AMPK), or the inhibition of mechanistic target of rapamycin complex 1 (mTORC1) initiates phagophore formation (Figure 2; Shang and Wang, 2011; Liu and Sabatini, 2020). The UPS can control the magnitude of the autophagy response via ULK1 and mTOR kinases (Liu C. et al., 2016; Nazio et al., 2016). Once autophagy is initiated, the phagophore membrane elongates and matures to become an autophagosome, which then fuses with a lysosome to form an autolysosome that degrades and recycles cargo (Figure 2).

Neurones are long-living, post-mitotic cells critically dependent on autophagy for maintaining their homeostasis and functionality (Stavoe and Holzbaur, 2019). Unsurprisingly, autophagy dysfunction is widely reported in ALS, AD, PD, and in aging. Neuronal cells are especially vulnerable to impaired autophagy—autophagosomes containing cargo to be degraded must be transported along the axon to the cell body to fuse with lysosomes, as lysosomes are rarely found in distal axons (Maday et al., 2012; Cheng et al., 2015). This vulnerability results in opportunity for proteins to aggregate and form pathological inclusions—a hallmark pathology of many NDs. A large body of evidence indicates MCFAs as a suitable intervention for combating symptoms of NDs and aging due in part to their autophagy-promoting effects. MCFAs are also known to inhibit autophagy when it is pathologically augmented, indicating that MCFAs may have a modulatory effect on autophagy in NDs, dependent on the context. One of the difficulties in reviewing the use of MCFAs in treating NDs, is the variety of different cell types and models used to study autophagy. We attempt to reconcile the dysregulation of autophagy in various model systems, and how the involvement of MCFAs affect autophagy.

### 4.1. MCFAs may promote healthy aging via upregulation of autophagy

Reduced autophagic activity is thought to be responsible for accumulating damaged or dysfunctional cellular components during aging, significantly contributing to the organismal functional decline. A study using *S. cerevisiae* to determine autophagy-related genes in aging identified several short-lived mutants as having defective autophagy (Matecic et al., 2010). Deletion or suppression of *Atg* genes can reduce the lifespan of various model organisms, and essential autophagy genes such as *Atg5* and *Atg7* are downregulated in the human brain during normal aging (Figure 2; Komatsu et al., 2005; Simonsen et al., 2008; Tóth et al., 2008; Lipinski et al., 2010).

Nutritional ketosis, brought upon by the MCT diet, is known to upregulate autophagy throughout the brain, via inhibition of mTORC1 (Table 2; McCarty et al., 2015). DA has been demonstrated to stimulate autophagy in *Dictyostelium*, a widely used model to study autophagic clearance, seen via the increase in autophagosome number and enhanced autophagic flux, likely due to increased expression of *Atg1* and *Atg8a* (Table 2; Mesquita et al., 2017; Warren et al., 2021). The term “autophagic flux” describes the dynamic process of autophagy from phagophore initiation to lysosome recycling and is often used as a measurement of degradation activity. Interestingly, these results were not seen with OA, suggesting that the effect on autophagy may be specific to DA (Warren et al., 2021). However, OA can stimulate c-Jun N-terminal kinase (JNK)-dependent autophagy in rats (Table 2; He et al., 2022). JNK activation is known to extend the lifespan in *Drosophila*, indicating that OA can promote healthy aging, albeit through a different mechanism to DA (Table 2; Wang et al., 2005). Furthermore, activation of JNK-dependent autophagy increases Beclin-1 expression, a key protein in autophagosome membrane elongation (Figure 2; Park et al., 2009) suggesting an mTOR-independent modulation of autophagy by OA.

In addition to its role in maintaining cellular homeostasis, autophagy has also been shown to have anti-inflammatory and antioxidant effects (Giordano et al., 2014; Qian et al., 2017). This suggests that a decline in autophagic activity may contribute to the chronic inflammation and oxidative stress associated with aging. Low grade, chronic inflammation is known to promote aging; referred to as “inflammageing,” this condition is characterized by increased levels of inflammatory markers throughout the body (Franceschi et al., 2000). The possible beneficial impact of MCFAs on aging may therefore stem from their autophagy-promoting effects, mediated by decreased inflammation and oxidative stress.

### 4.2. MCFAs could correct dysregulated autophagic flux in Alzheimer’s disease

Autophagy dysregulation is a common occurrence in AD. Early studies detected large amounts of aggregated tau protein and subcellular vesicles within swollen neurites (a common AD pathology) in AD patient brains; these vesicles were later identified to be immature autophagosomes (Suzuki and Terry, 1967; Nixon et al., 2005). Similar results were seen in AD mice, in which immature autophagosomes were shown to accumulate in neurites before β-amyloid (Aβ) plaques began to form, suggesting that autophagy dysfunction precedes, and possibly causes, pathological inclusion formation (Yu et al., 2005).

Medium-chain triglyceride diets have a positive effect on Aβ plaques, via the regulation of autophagy. The activity of HMGCS2, an enzyme that controls the synthesis of ketones from MCFAs, is mediated by the mTOR pathway (Hu et al., 2017). HMGCS2 induces the autophagic clearance of Aβ precursor protein (APP), thus reducing the occurrence of Aβ plaques (Table 2; Hu et al., 2017). Autophagy regulation by HMGCS2 is dependent on ketones, therefore, an MCT diet, or indeed MCFA supplementation, will increase the activity of HMGCS2 and clearance of APP (Table 2; Hu et al., 2017). Aβ accumulation is largely caused by the imbalance between its production and clearance (Selkoe and Hardy, 2016). It is plausible to assume that, by preventing the production of Aβ plaques, MCFA treatment may delay AD onset and progression.

Normal autophagic flux is essential for neuronal homeostasis (Zhang et al., 2013), and its impairment is known to correlate with AD progression (Chung et al., 2019). Proper formation and degradation of autophagosomes is essential for normal autophagic flux and the PI3K-CIII complex plays a vital role in autophagosome formation. The expression of Beclin-1, a core subunit of the PI3K-CIII complex, is significantly reduced in AD; consequently, the generation of PI3P, the product of PI3K-CIII complex activity, is also downregulated in the patients’ brains (Figure 2; Pickford et al., 2008; Lucin et al., 2013; Morel et al., 2013). A recent study showed that a diet high in MCFAs—achieved by replacing animal fat with coconut oil—can restore impaired autophagic flux in mouse hepatocytes (Wang et al., 2017). Interestingly, this functional restoration is independent of AMPK and mTOR signaling, suggesting that MCFAs, unlike short-chain fatty acids (Iannucci et al., 2016), may not act directly on the classic autophagy-regulating pathways (Wang et al., 2017). As MCFAs can restore normal autophagic flux, it is possible that treatment will slow down or even prevent disease progression in AD.

### 4.3. MCFAs may improve motor symptoms in amyotrophic lateral sclerosis by upregulating autophagy

The ketogenic diet has proven to be an effective therapy in mouse models of ALS by promoting mitochondrial energy production and membrane stabilization (Zhao et al., 2006). However, whether MCFAs influence autophagy in ALS remains unclear. Several ALS-associated genes are functionally implicated in autophagy, including *SQSTM1*, *C9orf72*, *Ubiquilin-2*, and *VAPB* (Mao et al., 2019; Şentürk et al., 2019; Wu et al., 2020; Cozzi and Ferrari, 2022). The C9orf72 protein binds GTPases Rab7, and Rab11 to aid endosome maturation and recycling, interacts with Atg1-ULK complex to aid autophagosome formation, and regulates autophagic flux via Rab8a and Rab39b (Sellier et al., 2016; Sullivan et al., 2016; Farg et al., 2017). Mutations in *C9orf72* impair autophagic flux, resulting in protein aggregation leading to motor neurone dysfunction and death (Sellier et al., 2016). Toxic dipeptide repeats aberrantly translated from the hexanucleotide repeat expansion region within intron 1 of the *C9orf72* gene disrupt the VAPB-PTPIP51 interaction at the mitochondrial-endoplasmic reticulum interface (Gomez-Suaga et al., 2022), thereby linking mitochondrial function to autophagy, through two ALS disease-causing genes, *C9orf72* and *VAPB*. When functioning normally, the autophagy receptor p62/SQSTM1 interacts with proteins targeted for degradation and brings them into the autophagosome where the receptor and its cargo are degraded upon fusion with a lysosome (Figure 2; Klionsky et al., 2016). The presence of p62/SQSTM1-positive inclusions in ALS patient motor neurones indicates that autophagic flux has been disrupted, as degradation of cargo has not been achieved (Al-Sarraj et al., 2011). As MCFAs are proven to restore autophagic flux in hepatocytes, it is possible that the same effect will be seen in motor neurones (Wang et al., 2017). Furthermore, MCFAs may also combat the decrease in autophagy seen in ALS. Rapamycin, a known inhibitor of mTORC1 and a potent autophagy inducer in many cell types including neurones (Noda and Ohsumi, 1998; Rangaraju et al., 2010), can rescue impaired locomotion in a zebrafish model of ALS (Table 2; Lattante et al., 2015). Preclinical data show that decanoic acid can inhibit mTORC1 in patient-derived astrocytes, much in the same way as rapamycin, resulting in an overall increase in autophagy (Table 2; Warren et al., 2020). These results suggest that MCFAs have the possibility to alleviate motor symptoms of ALS by increasing autophagy levels.

Due to their positive effects on both autophagy and mitochondrial function, MCFAs hold promise as a novel, multi-target therapeutic strategy for ALS. However, this may not be true for all types of ALS. Mouse models of ALS caused by mutations in the copper-zinc superoxide dismutase (SOD1) enzyme demonstrate pathological induction of autophagy in the spinal cord in the early, pre-symptomatic stages of the disease (Li et al., 2008; Zhang et al., 2011). When SOD1 ALS mice were treated with rapamycin, motor neurone degeneration was accelerated and the lifespan was significantly shortened (Zhang et al., 2011), providing evidence that stimulation of autophagy is not always beneficial in ALS. Clearly, the relationship between autophagy and neurodegeneration is complex, and a delicate balance of autophagy is likely required for health benefits in NDs.

### 4.4. MCFAs may modulate autophagy in Parkinson’s disease, and increase the clearance of pathological inclusions by promoting chaperone-mediated autophagy

Mutations in α-synuclein (*SNCA*) and *leucine-rich repeat kinase 2* (*LRRK2*, *PARK8*) are associated with genetic forms of PD, and have been widely studied for their involvement in autophagy dysfunction. The mechanisms by which these genes affect autophagic clearance and contribute to PD pathology are still being uncovered, but the upregulation of autophagy is a promising therapeutic intervention for PD.

Mutations in *LRRK2* are the most common genetic cause of PD. LRRK2 is a kinase that regulates many autophagy-related processes within a cell, including vesicular trafficking and endosomal transport (Dodson et al., 2012; Steger et al., 2016; Connor-Robson et al., 2019). Disease-causing *LRRK2* mutations increase the kinase activity of LRRK2, contributing to PD pathology (Jeong and Lee, 2020). LRRK2 is normally degraded in lysosomes via CMA; the G2019S mutation inhibits this process (Figure 2; Orenstein et al., 2013). BHB, one of the main ketones generated via MCFA metabolism, directly promotes CMA activation by increasing protein oxidation, resulting in increased clearance of proteins damaged by oxidation (Table 2; Finn and Dice, 2005). An activation of CMA by MCFAs may therefore increase the degradation of mutated LRRK2, preventing increased kinase activity (Table 2).

Interestingly, the G2019S mutation in *LRRK2* also increases autophagy in cultured human neurons, possibly to compensate for the reduction in CMA, resulting in neurite shortening—a pathological hallmark that precedes neuronal death in many NDs (Table 2; Plowey et al., 2008). It is possible that MCFAs are a suitable treatment to simultaneously promote CMA and decrease autophagy in PD patients. Fatty acids are known to inhibit autophagy regulated by AMPK activation in mice (Liu T. et al., 2016) and mTOR suppression in rat and human hepatocytes (Vinciguerra et al., 2008), it remains unclear, however, whether this is mediated by MCFAs. Treatment with lauric acid (LA), an MCFA with 12 carbon atoms, can decrease pathologically augmented autophagy in human chondrocytes (cells responsible for cartilage formation) as shown by the reduced expression of Atg5 and Beclin-1, proteins involved in the elongation and maturation of the autophagosome (Figure 2; Sekar et al., 2018). LCFAs such as palmitic acid, myristic acid and stearic acid further increased autophagy in chondrocytes, indicating that LA, and perhaps other MCFAs, may be better at modulating autophagy (Sekar et al., 2018). LCFAs also induced the expression of LC3, a key player in autophagosome formation, whereas LA treatment returned LC3 expression to the control levels, suggesting stabilization of autophagosome production (Sekar et al., 2018). On the other hand, a recent study demonstrated that LA could induce autophagy in wild-type pig intestinal cells, resulting in dysregulated autophagic flux and cell death (Yang et al., 2020). These studies show that LA can promote or reduce autophagy depending on the context, indicating LA may be an ideal treatment to normalize autophagy homeostasis in ND patients (Table 2).

Accumulation of α-synuclein is a hallmark pathology in both sporadic and familial PD. Although the normal function of α-synuclein has not been fully elucidated, it is thought that this protein plays a role in the formation of synaptic vesicles (Burré et al., 2010). The A53T mutation in *SNCA* has been linked to autophagy; cells expressing A53T mutant α-synuclein display accumulation of autophagic vesicles, suggesting an impairment of autophagic flux (Stefanis et al., 2001). Like LRRK2, α-synuclein is also degraded via CMA; A53T mutations prevent this CMA-associated degradation and increase autophagy, likely as a compensatory response (Table 2; Cuervo and Dice, 2000). Considering the autophagy-stimulating activity of MCFAs, it is possible that this class of lipids may promote the degradation of α-synuclein via an increase in CMA (Finn and Dice, 2005). LA treatment may also be successful in ameliorating the compensatory increase in autophagy, restoring autophagic flux (Sekar et al., 2018; Yang et al., 2020). Overall, it seems that MCFA treatment may be advantageous in both sporadic and familial PD.

## 5. Discussion

Medium chain fatty acids have a direct role in regulating autophagy and mitochondrial function, processes that are dysfunctional in aging and NDs. This review highlights evidence that MCFAs have the potential to be sufficient treatments for some NDs, and promote healthy aging, due to their effects on autophagic processes and the function of mitochondria. It is possible that MCFAs affect dysregulated autophagy and mitochondrial function together as well as in parallel, providing more evidence that they are an advantageous treatment option. It is worth noting that there is an overlap of disease-causing genes involved various neurodegenerative diseases. Thus, MCFA treatments that modulate the severity of AD and PD, may also be effective in treating ALS and possibly other neurodegenerative diseases. However, this is an under-researched area and there are currently very few studies reporting a link between MCFAs and autophagy and mitochondrial function in neurones. Adverse side effects associated with MCFAs and the MCT diet have also been reported, warranting further studies into the mode of action and safety aspects of these compounds.

### 5.1. Avenues for future research and limitations of research into MCFAs

A systematic analysis of each MCFA using animal models will be required to gain a clearer picture of the effects that these compounds have on different neurodegenerative diseases. Drosophila would be particularly amenable to this type of analysis as the disease models are well established, and tissue-specific effects as well as whole organism effects can be easily determined. In the case of ALS, Drosophila could be used to assess differences between motor neurone and brain degeneration as neural cell type-specific toolsets are readily available to the research community (Jenett et al., 2012; Aso et al., 2014).

In addition, modifications to individual MCFAs may provide compounds with more specific biological effects. For example, 4-MOA and 5-MOA are both methyl-modified derivatives of octanoic acid, and it would be interesting to compare the different effects that these molecules have on neuronal biology at the molecular level.

One of the limitations of the research into the biological effects of MCFAs is the lack of a standardized model that can be used to compare the effects of different compounds on neural function. As a result, many of the observations are specific to a given cell, or a given organism. Given that Drosophila has both motor neurones and a complex brain, this model appears ideally positioned for studying the effects of MCFAs on tissue specific and organ-specific alterations. In addition, the complete neural circuitry of the fly brain has recently been mapped (Winding et al., 2023), aiding the efforts to understand how MCFAs modulate neurodegeneration at the level of neurons and connecting synapses.

## Author contributions

ED and BZ wrote the manuscript, summarized literature, and prepared figures. VKS wrote and revised the manuscript. HA conceptualized and designed the article and wrote and revised the manuscript. All authors contributed to the article and approved the submitted version.
